# Tuning Design Parameters of ICAM-1-Targeted 3DNA Nanocarriers to Optimize Pulmonary Targeting Depending on Drug Type

**DOI:** 10.3390/pharmaceutics14071496

**Published:** 2022-07-19

**Authors:** Nikša Roki, Melani Solomon, Jessica Bowers, Lori Getts, Robert C. Getts, Silvia Muro

**Affiliations:** 1 Fischell Department of Bioengineering, University of Maryland, College Park, MD 20742, USA; roki.niksa@gmail.com; 2 Institute for Bioscience and Biotechnology Research, University of Maryland, College Park, MD 20742, USA; melani_solomon@yahoo.com; 3 Genisphere, Hatfield, PA 19940, USA; jessicabowers333@gmail.com (J.B.); lgetts@codebiotx.com (L.G.); bgetts@codebiotx.com (R.C.G.); 4 Code Biotherapeutics, Hatfield, PA 19940, USA; 5 Institute for Bioengineering of Catalonia of the Barcelona Institute of Science and Technology, 08028 Barcelona, Spain; 6 Institute of Catalonia for Research and Advanced Studies, 08010 Barcelona, Spain

**Keywords:** 3DNA nanocarrier, ICAM-1, lung targeting, carrier design parameters, multiparametric hierarchy, drug type

## Abstract

3DNA holds promise as a carrier for drugs that can be intercalated into its core or linked to surface arms. Coupling 3DNA to an antibody targeting intercellular adhesion molecule 1 (ICAM-1) results in high lung-specific biodistributions in vivo. While the role of individual parameters on ICAM-1 targeting has been studied for other nanocarriers, it has never been examined for 3DNA or in a manner capable of revealing the hierarchic interplay among said parameters. In this study, we used 2-layer vs. 4-layer anti-ICAM 3DNA and radiotracing to examine biodistribution in mice. We found that, below saturating conditions and within the ranges tested, the density of targeting antibodies on 3DNA is the most relevant parameter driving lung targeting over liver clearance, compared to the number of antibodies per carrier, total antibody dose, 3DNA dose, 3DNA size, or the administered concentration, which influenced the dose in organs but not the lung specific-over-liver clearance ratio. Data predicts that lung-specific delivery of intercalating (core loaded) drugs can be tuned using this biodistribution pattern, while that of arm-linked (surface loaded) drugs requires a careful parametric balance because increasing anti-ICAM density reduces the number of 3DNA arms available for drug loading.

## 1. Introduction

DNA has emerged as a new material for drug delivery, a field where it holds considerable potential [1,2]. DNA-made nanocarriers (DNA-NCs) are amenable to recombinant or synthetic manufacturing, self-assemble with exceptional reproducibility and architectural versatility, and can be tuned to control their bioactivity and degradation [1,3,4,5]. Targeting moieties, imaging agents, and therapeutics can be incorporated in a variety of ways [4,6,7], and responsiveness to physiological cues (pH, temperature) can be integrated for release on-demand [1]. These systems are explored for drug delivery to extracellular or intracellular compartments, including the endo-lysosomal system, cytosol, or nucleus [5,8]. Their site-specific delivery to certain cells and tissues can be achieved by functionalization with targeting moieties that recognize particular cell-surface markers, e.g., transferrin receptor, HER2, folic acid receptor, mucin 1, and cell adhesion molecules (CAMs) [8,9,10,11]. Examples of DNA-NCs include spherical DNA, origami, cages, dendritic structures, liposomal and tubular designs, etc. [1,12]. Yet, there is little characterization of the interaction of DNA-NCs with biological systems, particularly in vivo, and the modulation of design parameters to specifically tune this interaction.

**Figure 1:**
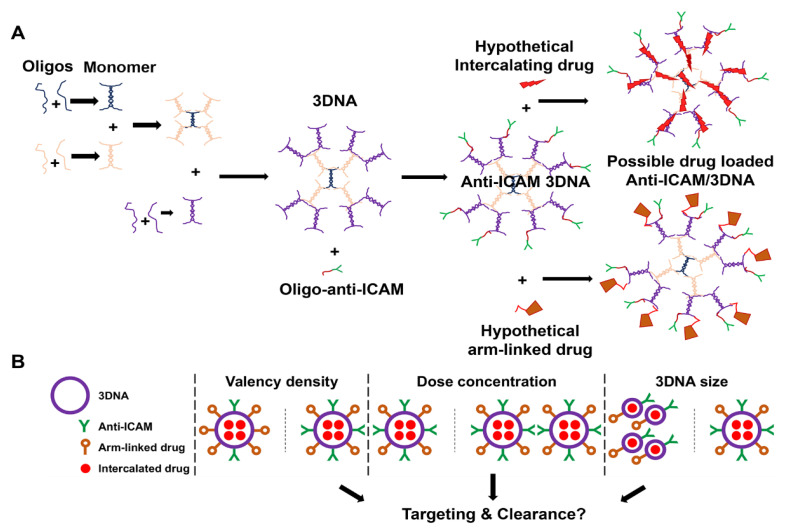
Anti-ICAM/3DNA synthesis and parametric variables. (**A**) DNA oligonucleotides (oligos) hybridize in pairs generating monomers that are assembled by hybridization layer-by-layer into 3DNA. Anti-ICAM antibody-oligo conjugate is hybridized with 3DNA whose outer arms are complementary in sequence, resulting in anti-ICAM/3DNA nanocarriers. Theoretically, drugs can be either intercalated within the double-stranded DNA regions or linked to single-stranded arms, e.g., by conjugating them to complementary oligos. (**B**) The goal of this study was to examine the influence of the 3DNA parameters depicted on the in vivo biodistribution of anti-ICAM/3DNA, not only their individual role, but for the first time, their hierarchical interplay, including targeting valency density (antibody per nanocarrier surface area), dose concentration, and size, along with additional parameters that intrinsically vary with these parameters, such as 3DNA per kg of body weight, antibody per kg, absolute number of antibody molecules per nanocarrier regardless of 3DNA size, etc.

3DNA^®^ is a good model DNA-NC to study and characterize in vivo biodistribution and drug delivery. 3DNA is a tri-dimensional DNA nanoscaffold of highly branched architecture, assembled via layer-by-layer hybridization of small DNA subunits based on sequence complementarity, to specifically control and precisely tune the final NC size and number of peripheral arms (Figure 1A) [6,13,14]. Psoralen is used to crosslink the internal double-stranded DNA regions for stability [13,15]. Single, various, or all 3DNA terminal arms can be functionalized with therapeutic, targeting, and/or imaging agents whose quantity, combination, and spatial distribution can be controlled by sequence. 3DNA has long been used as a signal amplifier in biomolecular applications under the name, 3DNA^®^ UltraAmp [15]. After the first demonstration of its potential for targeted drug delivery [16], several studies have validated 3DNA to deliver toxins, proteins, and genetic materials in diverse cell types (endothelial, epithelial, mesothelial, fibroblasts, etc.) [6,8,14,17], by utilizing various cell-surface markers, such as intercellular adhesion molecule 1 (ICAM-1), platelet-endothelial cell adhesion molecule 1 (PECAM-1), transferrin receptor, mannose-6-phosphate receptor, folate receptor, etc. [6,8,14,17]. Examples of applications under investigation include plasmid delivery in a mouse model of pancreatic cancer [17], siRNA delivery in a mouse model of ovarian cancer [14], peptide delivery in a mouse model of preterm brain injury [18], and doxorubicin delivery in models of secondary cataracts using human lens tissue explants [6] and rabbits [19].

In particular, 3DNA was shown to achieve one of the highest levels of targeting specificity found in the literature for an in vivo model, obtained by targeting ICAM-1 [7]. ICAM-1 is a protein found primarily on endothelial cells and overexpressed in pathologies characterized by inflammation [20]. Several groups are exploring ICAM-1 targeting for interventions in cardiovascular diseases, cancer, genetic conditions, lung maladies, etc. [20,21,22,23,24,25,26]. Due to high expression in the pulmonary endothelium and the fact that the lungs receive the full cardiac output after intravenous (IV) administration, this organ represents a privileged site for ICAM-1 targeting [20]. Coupling anti-ICAM antibody (Ab) to 3DNA (anti-ICAM/3DNA) resulted in a 424-fold specific targeting increase over non-specific IgG/3DNA (specificity index; see Section 2) [7].

The goal of this study (Figure 1B) was to explore how varying 3DNA design parameters would impact in vivo targeting and biodistribution to guide future delivery of drugs to the lungs alone (for pulmonary conditions) or to the lungs and other organs (for systemic or spread disease). Drugs may be loaded in 3DNA by two means (Figure 1A): (1) intercalation within the DNA scaffold (inner loading) or (2) coupling to peripheral 3DNA arms (surface loading) by annealing complementary oligonucleotide-drug conjugates or by direct chemical linkage of the drug [1]. Many anti-cancer drugs intercalate within DNA, such as anthracyclines (e.g., doxorubicin), dactinomycin, or mitoxantrone [27]. These compounds contain planar aromatic or heteroaromatic rings which fit in the hydrophobic space between adjacent DNA base pairs [27]. Hence, anti-ICAM/3DNA could be a good NC to deliver these intercalated compounds for lung cancer or other diseases. Other therapeutic molecules directly conjugated to 3DNA outer arms or hybridized to outer arms as oligonucleotide conjugates [1] may include antioxidant enzymes for acute lung injury [28], thrombolytic agents for pulmonary embolism [29], lysosomal enzyme replacement therapy for pulmonary Niemann–Pick disease type B [21], and nucleic acids for gene therapy with siRNA, microRNA, CRISPR, etc. [30]. While in the present study we did not load 3DNA with any specific drug, the precise assembly of this NC (Figure 1A) provides reliable information on the number of 3DNA arms available for drug linkage as well as intercalating sites. Therefore, knowing 3DNA biodistribution in the body is valuable in estimating the expected biodistribution of drugs that can be loaded by these two means.

The modulation of drug NC properties including size, concentration, and number of targeting moieties (targeting valency) [2,31] plays a key role in their biodistribution [10,22,32,33,34,35]. Although few studies have examined the influence of these factors for DNA-NCs, results illustrate their relevance. The concentration dependence of targeting and/or cargo activity has been shown for tetrahedral and nanotube DNA-NCs addressed to the folic acid receptor in cancer cells and, for the tetrahedral design, in a mouse model [2,10,36]. The role of the targeting valency of DNA-NCs has been examined using similar formulations addressed to the folic acid receptor or nucleolin [2,31,36]. However, most of these studies were conducted in cellular models [3,4,5,8,9,10,11] or did not look into how such variations modulated targeting per se, but rather measured the activity obtained from the delivered cargo (siRNA) [6], which depends on additional functions (cytosolic release, etc.) [1]; hence, systematic in vivo targeting data is still scarce [7]. Similarly, although the literature often shows studies on such parametric influence on the biodistribution of drug carriers, including ICAM-1 targeting ones [32,33,35], whether such a role similarly applies across different formulations and, most importantly, the comparative level of influence or hierarchy among different design parameters has never been examined in vivo.

In addition, for 3DNA, these design parameters are expected to influence not only targeting and body distribution but also the loading of intercalating or arm-linked dugs [1,6]. Increasing the number of antibodies occupying 3DNA outer arms may favor specific targeting but reduce the number of therapeutic molecules that can be linked to the remaining arms [1,6,7]. If a drug is incorporated by DNA intercalation, then its delivery would depend on the amount of DNA scaffold present [1,6] and tuning the targeting valency would not interfere with drug loading but would influence biodistribution. Instead, varying the size of 3DNA would impact the targeting and biodistribution of both inner-loaded drugs and surface-loaded drugs. Therefore, the biodistribution resulting from varying all of these 3DNA parameters is difficult to predict and must be empirically examined.

Such a characterization was the goal of the present study. We used anti-ICAM/3DNA formulations of varying size, targeting valency (antibody molecules per NC surface area), and dose concentration to examine the simultaneous and hierarchical influence of these parameters on NC biodistribution in vivo (Figure 1B), along with parameters that intrinsically vary with these ones, such as 3DNA per kg of body weight, antibody per kg, absolute number of antibody molecules per nanocarrier regardless of 3DNA size, etc. This is the first time that the impact of design and administration parameters of a DNA-based NC is studied respective to its in vivo biodistribution. It is also the first time that multiple design and administration parameters are simultaneously compared to decipher their hierarchical role and obtain the most influencing parameter among them all. Finally, it is the first time that NC biodistribution along with known NC architecture are used to estimate in silico the influence of these parameters on the biodistribution of drugs, depending on whether they would be intercalated into or arm-linked to 3DNA, showing a differential pattern. The data obtained will guide future applications of drug delivery using 3DNA.

## 2. Materials and Methods

### 2.1. Antibodies and Reagents

Rat anti-mouse ICAM-1 (anti-ICAM) antibody YN1, was produced from respective hybridoma from the American Type Culture Collection (Manassas, VA, USA). Non-specific IgG was from Jackson Immunoresearch (Pike West Grove, PA, USA). 5′-modified DNA oligonucleotide (72-mer) was from Oligo Factory (Holliston, MA, USA). Pierce bond-breaker TCEP solution, LC-SMCC crosslinker, 7 k MWCO Zeba spin columns, thiophilic adsorption resin, heterobifunctional Pierce crosslinking kit, bovine serum albumin (BSA), and trichloroacetic acid (TCA) were from Fisher Scientific (Kerrville, TX, USA). Rabbit anti-mouse PECAM-1 antibody was from Novus Biologicals (Centennial, CO, USA) and FITC-conjugated goat anti-rabbit IgG was from Invitrogen (Carlsbad, CA, USA). Iodogen iodination tubes were from Pierce (Rockford, Illinois) and BioSpin Tris Columns were from BioRad (Hercules, California). Amicon 10 kDa MWCO spin filters were from Millipore Sigma. All other reagents were from Sigma Chemical (St. Louis, MO, USA).

### 2.2. Preparation of Anti-ICAM/3DNA NCs

First, 3DNA was manufactured by Genisphere LLC as published [13]. Briefly, DNA oligonucleotides (oligos) were synthesized, which hybridized in pairs, generating monomers that contained a waist region of double-stranded DNA and single-stranded arms. The sequences of arms are such that modules assembled layer-by-layer by hybridization to the arms of other modules, forming a tridimensional structure called 3DNA (Figure 1A). The sequence of the oligos used to this end is not provided as it represents Code Biotherapeutics proprietary information. The process was stopped so that the final number of layers was 2 or 4 (2L or 4L 3DNA), with an average MW of 1200 or 11,000 kDa and 36 or 324 peripheral arms, respectively.

Then, 3DNA was linked to an Ab targeting ICAM-1 (anti-ICAM) or a non-specific Ab (IgG) by conjugating them, via NHS-maleimide chemistry, to oligos whose sequence was complementary to that of 3DNA outer arms, and then mixing oligo-conjugated Ab and 3DNA, as published [7]. Briefly, Ab was reacted with LC-SMCC and then excess crosslinker was removed using Zeba spin columns. In parallel, a 5′-thiol-modified 72-mer DNA oligo was reduced in TCEP as described [7]. The resulting Ab-oligo conjugate was concentrated using Amicon 10 kDa MWCO spin filters and then annealed by complementarity to 2L or 4L 3DNA by incubation at 37 °C for 30 min with a Tm of 72 °C [7]. The sequence of the oligo used for this purpose is not provided as it represents Code Biotherapeutics proprietary information.

Precise Ab surface densities were pursued by hybridizing 3DNA with Ab-oligo in the desired molar ratios (Table 1). Non-specific IgG/3DNA formulations were used as controls (targeting valency = 0; Table 1). The average diameter, polydispersity index (PDI), and ζ-potential of Ab/3DNA were measured by dynamic light scattering (DLS) and electrophoretic mobility using the Malvern Zetasizer (Worcestershire, UK), as described [7]. The number of Ab molecules on 3DNA was determined using Ab-oligo conjugates that were further coupled to ^125^Iodine (^125^I) prior to their annealing to 3DNA outer arms (see Section 2.3 below). The fraction of ^125^I-Ab-oligo that was not annealed to 3DNA was removed by filtration through a 1000 kDa filter, followed by quantification of ^125^I-Ab-oligo annealed to 3DNA in a gamma counter (PerkinElmer, Boston, MA, USA), as described [7].

### 2.3. ^125^Iodine Labeling of Antibody-Oligonucleotide Conjugates

Where indicated, anti-ICAM-oligo or IgG-oligo were labeled with ^125^I or Cy3 for radioisotopic quantification or fluorescence tracing, respectively. Conjugation to ^125^I was achieved by incubating 1 mg/mL Ab-oligo for 5 min at 4 °C with 20 µCi ^125^I, using Pierce Iodogen, after which non-conjugated ^125^I was removed using BioSpin Tris Columns (BioRad; Hercules, CA, USA). The specific activity (cpms/mass) of the resulting ^125^I-Ab-oligo was obtained by further separating free ^125^I by precipitation in 15% (*v*/*v*) trichloroacetic acid (TCA), followed by centrifugation to separate ^125^I-Ab-oligo in the pellet and quantification of respective cpms and protein concentration, as published [7].

Fluorescent labeling of 3DNA was pursued using commercial Cy3-oligo conjugates from Integrated DNA Technologies (Coralville, IA, USA), where the fluorophore was located at the 5′-end of an oligo whose sequence was complementary to that of 3DNA arms. The conjugate was hybridized to 3DNA, followed by psoralen crosslinking and purification using size exclusion chromatography. Ab-oligo was then annealed to Cy3-3DNA as described above in Section 2.2.

### 2.4. Biodistribution of Anti-ICAM/3DNA in Mice

Anesthetized C57BL/6 mice were injected IV with the ^125^I-Ab/3DNA formulations shown in Supplementary Table S1. These formulations had different sizes (2L and 4L), targeting valencies (6–80 Ab per NC; 143–1273 Ab per µm^2^ of 3DNA surface area), and dose concentrations (40–400 µg DNA per kg of body weight or BW), as indicated in respective figure legends. Injections of control 3DNA or non-specific IgG/3DNA (targeting valency 0) encompassed 400 µg 3DNA/kg BW and ~500–550 Ab/µm^2^ for the IgG/3DNA formulation. Several permutations of 2L and 4L Ab/3DNA were compared, whose respective parameters are described in Supplementary Table S1. Blood was collected from the retro-orbital sinus at 1, 5, 15, 30, and 60 min post-injection, and organs were collected at 60 min, after sacrifice. Samples were weighed, homogenized at 28,000 rpm using Kinematica Polytron^TM^ PT 3100D (Kinematica, Lucerne, Switzerland), and incubated for 15 min at 4 °C with TCA followed by centrifugation to eliminate any free ^125^I in the supernatant, as described [37]. Radioactivity measurements using a gamma counter were utilized to calculate the percentage of injected dose in the blood and each organ (%ID), where the injected dose is the dose measured prior to injection minus the dose remnant in the syringe after the injection (Equation (1)). We also calculated the %ID per gram of organ (%ID/g) to compare the “concentration” of Ab/3DNA reached in each organ (Equation (2)). This parameter is shown in the main figures, while the respective %ID is shown in Supplementary Tables. The localization ratio was also calculated (LR = %ID/g in an organ ÷ %ID/g in blood; Equation (3)) to express the tissue-to-blood distribution, and the specificity index (SI = LR of a targeted formulation ÷ LR of the non-targeted formulation; Equation (4)) to estimate the targeting advantage [7]. Using biodistribution data and taking into account the known number of intercalating sites in each anti-ICAM/3DNA formulation and the respective number of free 3DNA arms (Supplementary Table S1), we calculated the number of “intercalating sites” and “free arms” for the amount of anti-ICAM/3DNA present in each organ.

(1) %ID=Dosepriortoinjection − Doseremnantinthesyringeafterinjection

(2) $$\%\mathrm{ID}/g=\frac{\%\mathrm{ID}}{\mathrm{gram}\mathrm{of}\mathrm{organ}}$$

(3) $$\mathrm{LR}=\frac{\frac{\%\mathrm{ID}}{g}\mathrm{in}\mathrm{an}\mathrm{organ}}{\frac{\%\mathrm{ID}}{g}\mathrm{in}\mathrm{blood}}$$

(4) $$\mathrm{SI}=\frac{\mathrm{LR}\mathrm{antiICAM/3DNA}}{\mathrm{LR}\mathrm{IgG/3DNA}}$$

### 2.5. Visualization of Lung Targeting of Anti-ICAM/3DNA in Mice

Cy3-labeled 4L 3DNA hybridized with either anti-ICAM-oligo or IgG-oligo conjugates (46 Ab molecules/NC; Supplementary Table S1) were injected IV in anesthetized C57/BL6 mice, as described above. Five min after injection and still under anesthesia, mice were perfused through cardiac puncture with phosphate buffer saline (PBS) to flush out the circulating blood, followed by perfusion with 4% paraformaldehyde to fix tissues. The lungs were then collected, fixed in 4% paraformaldehyde for an additional 48 h in the dark, and processed using formalin-fixed paraffin embedding and sectioning using Histoserv Inc. (Germantown, MD, USA). Lung sections were de-paraffinized, rehydrated with PBS, and antigen retrieval was performed by two 5 min cycles consisting of incubating the sample in a microwave at 800 W power in sodium citrate buffer containing 0.05% Tween-20 at pH 6.0. Tissue sections were then blocked for 30 min at room temperature in a solution containing 5% bovine serum albumin and 0.02% Triton X-100. Finally, samples were immunostained using DAPI to stain cellular nuclei, along with polyclonal anti-PECAM-1 and FITC-labeled secondary Ab, to localize formulations with the vascular endothelium. Sections were imaged using an LSM 710 confocal laser scanning microscope with 20× Plan-APOCHROMAT objectives and 405, 488, and 555 lasers (Zeiss; Oberkochen, Germany).

### 2.6. Ethical Use of Laboratory Animals

All animal experiments described in this article were officially approved and in compliance with all regulations. Please, see the Institutional Review Board Statement before the References section for complete information on this item.

### 2.7. Statistical Analysis

Data were calculated as mean ± standard error of the mean (S.E.M.), with *n* ≥ 5 mice being used for Ab/3DNA, and *n* ≥ 3 for controls consisting of 3DNA alone or non-specific IgG/3DNA. Significance was determined using either ANOVA followed by Tukey’s test for comparisons among more than two groups, or Student’s *t*-test when comparing two groups, assuming a *p*-value of 0.05.

## 3. Results and Discussion

### 3.1. Role of Targeting Valency and Dose Concentration on the Biodistribution of 4-Layer Anti-ICAM/3DNA

We first examined the effect of the targeting valency on the biodistribution of 4-layer (4L) anti-ICAM/3DNA, recently shown to achieve high lung specificity in mice [7]. 3DNA was mixed at various molar ratios with an anti-ICAM-oligonucleotide (Ab-oligo) conjugate with a sequence complementary to 3DNA outer arms (see Section 2.2), rendering 80, 46, 13, or 0 anti-ICAM molecules per NC, wherein valency 0 was the non-specific IgG/3DNA control (Table 1). These formulations showed similar mean hydrodynamic diameter (160–197 nm), polydispersity index (PDI 0.18–0.25), and ζ-potential (−39 to −45 mV) [7].

**Figure 2:**
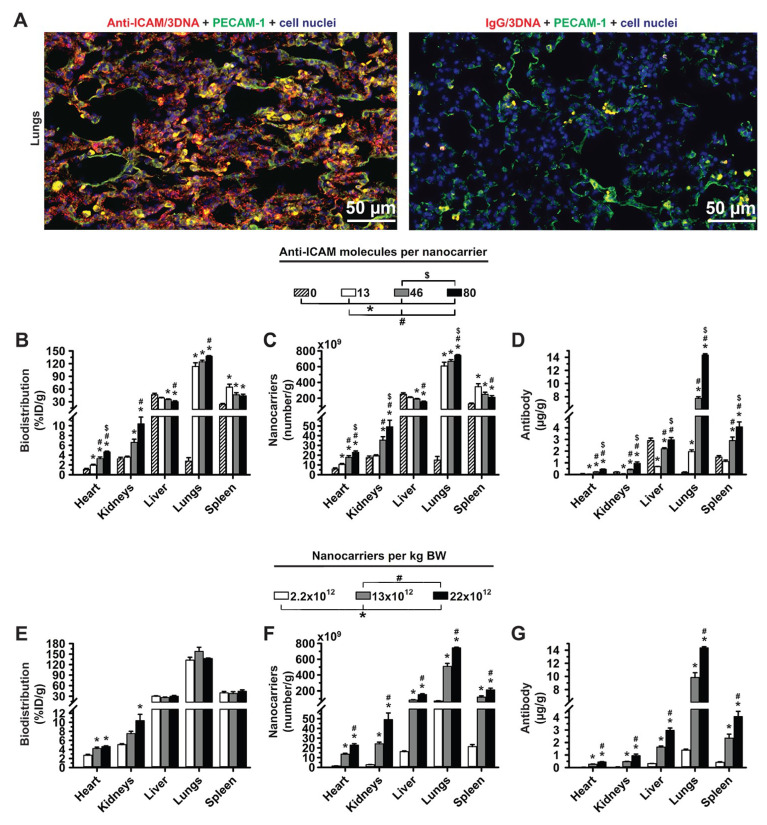
Role of targeting valency and dose concentration of the biodistribution of 4L anti-ICAM/3DNA. (**A**) Control IgG/Cy3-3DNA or anti-ICAM/Cy3-3DNA (Cy3 = red) were IV injected into C57BL/6 mice. Lungs were isolated upon sacrifice at 5 min, processed for confocal microscopy, and endothelial cells visualized using polyclonal anti-PECAM-1 + FITC-secondary antibody (green). (**B**–**D**) Mice were injected with 4-layer ^125^I-anti-ICAM/3DNA bearing different targeting valencies (valency 0 = IgG/3DNA control), or (**E**–**G**) at different dose concentrations. Organs’ radioactive content and weight were determined at 60 min to calculate: (**B**,**E**) % injected dose per gram of organ (% ID/g); (**C**,**F**) the number of nanocarriers per gram of organ; and (**D**,**G**) the antibody mass per gram of organ. Data are mean ± S.E.M. (n ≥ 3). * Compares formulations with (**B**–**D**) 0 anti-ICAM/NC or (**E**–**G**) 2.2 × 10^12^ NCs/kg BW to those with higher valency or dose, respectively. # Compares formulations with (**B**,**C**,**D**) 13 anti-ICAM molecules/NC or (**E**–**G**) 13 × 10^12^ NCs/kg BW to those with greater valency or dose. $ Compares formulations with (**B**–**D**) 46 anti-ICAM molecules/NC to those with greater valency; (*p* < 0.05).

As shown in Supplementary Table S2, all anti-ICAM/3DNA formulations disappeared quickly from the circulation, which was faster for higher valencies, likely due to fast targeting to ICAM-1 expressed on the surface of blood vessels. Indeed, fluorescence microscopy showed abundant anti-ICAM/3DNA colocalizing with PECAM-1-positive endothelium throughout the lungs (Figure 2A), the main target for ICAM-1 [7]. The higher the valency of anti-ICAM/3DNA, the higher the detection in the lungs (Figure 2B; notice that %ID/g is a concentration-like parameter; respective %ID is shown in Supplementary Tables S2 and S3). This was specific compared to control IgG/3DNA (targeting valency 0). Contrarily, increasing the targeting valency of anti-ICAM/3DNA resulted in lower concentrations in the liver and spleen (Figure 2B). Since ICAM-1 is expressed on the endothelium in these organs [37], the heart and kidneys also received increasing levels of anti-ICAM/3DNA with increasing targeting valencies, yet these levels were much lower than those in the lungs, liver, and spleen, and will not be discussed hereafter.

Increasing the targeting valency of anti-ICAM/3DNA enhanced the concentration of the NC component in the lungs and reduced it in the liver and spleen (Figure 2C), although these changes were not large in the conditions tested. Targeting valency more acutely affected the concentration of the Ab component (Figure 2D). Unlike the NC concentration, which decreased in the liver and spleen with increasing valency, anti-ICAM concentration increased in these organs. This can be explained as increasing valency from 13 to 80 only modestly reduced the %ID/g in these clearance organs (Figure 2B), yet each NC carried a very different number of anti-ICAM molecules (from 13 to 80).

Next, we focused on the role of the concentration of anti-ICAM/3DNA to be injected (dose concentration). Increasing this parameter lowered the circulating levels (Supplementary Table S2), yet, after 5 min no differences were observed, suggesting that targeting may have been achieved. In fact, varying the dose concentration did not impact the proportion of the injected dose per gram of lung, liver, or spleen (Figure 2E), although it enhanced the concentration of both NCs and Ab reaching the lungs (Figure 2F,G). This also enhanced the NC and Ab concentration in the liver and spleen.

Summarizing, increasing the targeting valency of 4L anti-ICAM/3DNA increased biodistribution to the lungs (target) and decreased biodistribution to the liver and spleen (off-target) based on %ID/g (Figure 2B) while varying the dose concentration (Figure 2E) did not seem to change biodistribution in a similar manner. This is different from previously described results on model polystyrene nanoparticles targeted to ICAM-1, for which the opposite tendency was observed [32]. The discrepancy may be due to the different formulations and parametric ranges used. For instance, this study compared 4L anti-ICAM/3DNA bearing 143–879 Ab molecules per µm^2^ of NC surface, while previous anti-ICAM polymeric NCs varied between 1750–6600 Ab molecules/µm^2^. Such a high targeting valency of polymeric NCs may have been closer to saturation so that increasing this parameter had a lesser impact on the specific lung-over-liver targeting compared to 4L anti-ICAM/3DNA. Another difference is that anti-ICAM had been surface-adsorbed on polymeric nanoparticles while in this study it was oligo-annealed to 3DNA branches. It is possible that not all adsorbed Abs may engage in specific receptor binding, while more flexible 3DNA branches may favor ICAM targeting so that this parameter may influence more profusely the resulting biodistribution.

As per the NC concentration in organs, changing the targeting valency of 4L anti-ICAM/3DNA seemed advantageous over changing dose concentration in order to achieve lung specificity (Figure 2C vs. Figure 2F). This was not the case for Ab concentration (Figure 2D vs. Figure 2G), which followed a similar trend in both cases: increasing targeting valency or dose concentration both resulted in enhanced Ab concentration in lung and liver–spleen. Hence, when increasing the targeting valency or dose concentration, one must consider whether the increase in formulation uptake by the target organ, the lungs, compensates for the higher liver–spleen uptake of the Ab component. Depending on the application intended, these adjustments may cause side effects in clearance organs (if anti-ICAM would prevent the positive influence of ICAM-1 in certain conditions) or beneficial effects (if anti-ICAM would block the negative influence of ICAM-1 in other conditions), as both are possible based on the literature [38,39].

### 3.2. Significance of Targeting Valency and Dose Concentration of 4L Anti-ICAM/3DNA for Intercalating vs. Arm-Linked Drugs

**Figure 3:**
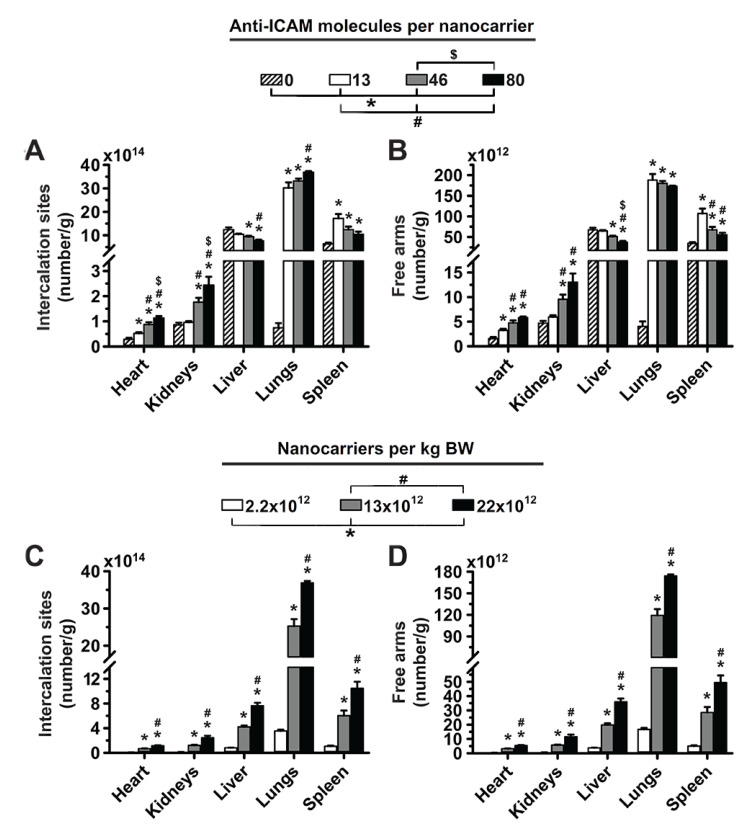
Effect of 4L anti-ICAM/3DNA targeting valency and dose concentration on the potential biodistribution of intercalating and arm-coupling drugs. 4-layer ^125^I-anti-ICAM/3DNA were IV injected in C57BL/6 mice at (**A**,**B**) different targeting valencies, or (**C**,**D**) different dose concentrations. Organs’ radioactive content and weight were determined at 60 min to calculate: (**A**,**C**) the number of DNA drug-intercalating sites per gram of organ; and (**B**,**D**) the number of outer arms free for drug coupling per gram of organ. Data are mean ± S.E.M (n ≥ 3). * Compares formulations with (**A**,**B**) 0 anti-ICAM/NC or (**C**,**D**) 2.2 × 10^12^ NCs/kg BW to those with higher valency or dose, respectively. # Compares formulations with (**A**,**B**) 13 anti-ICAM molecules/NC or (**C**,**D**) 13 × 10^12^ NCs/kg BW to those with greater valency or dose. $ Compares formulations with (**A**,**B**) 46 anti-ICAM molecules/NC to those with greater valency; (*p* < 0.05).

Next, we estimated how the biodistribution of anti-ICAM/3DNA would impact the biodistribution of drugs that 3DNA could potentially carry. Loading of an intercalating drug in 3DNA (inner loading) is known to depend on the DNA content. Based on experience, Code Biotherapeutics estimates a loading capacity of 550 doxorubicin molecules per 2L 3DNA and 4950 doxorubicin molecules per 4L 3DNA (unpublished). We used this information to plot the biodistribution of an intercalating drug, expressed as the number of effective intercalation sites per gram of tissue (Figure 3A). On the other hand, the loading of an arm-linked drug (surface loading) is known to depend on the number of 3DNA arms available for coupling (not occupied with targeting Ab). Thus, we used this information to plot the biodistribution of an arm-linked drug (Figure 3B). As expected, increasing the targeting valency of anti-ICAM/3DNA enhanced lung concentration of drug-intercalating sites and decreased that in the liver and spleen (Figure 3A). However, this decreased the concentration of free arms available for drug loading in the lungs, liver, and spleen (Figure 3B), signifying that the targeting/non-targeting gain obtained from increasing valency may not compensate for the loss of NC arms available for drug coupling. Additionally, increasing the concentration of anti-ICAM/3DNA in the injected dose enhanced the concentration of both drug-intercalating sites and drug-coupling arms in the lungs, liver, and spleen (Figure 3C,D). Thus, within the range tested, the advantage of increasing anti-ICAM/3DNA valency seems more valuable for lung targeting of intercalating vs. arm-linked drugs, while increasing the dose concentration impacts both drug types similarly.

### 3.3. Role of Targeting Valency and Dose Concentration on the Biodistribution of 2-Layer Anti-ICAM/3DNA

We then determined the biodistribution of smaller 2-layer (2L) formulations. As shown in Table 1, IgG and anti-ICAM formulations with different targeting valencies had a similar mean hydrodynamic diameter (113–120 nm diameter), PDI (0.29–0.35), and ζ-potential (−35 to −39 mV). Since this was our first time using 2L anti-ICAM/3DNA, we verified lung targeting. As with 4L, 2L anti-ICAM/3DNA disappeared fast from the circulation (Supplementary Figure S1), although the blood level after 5 min was slightly higher vs. 4L formulations (Supplementary Table S2). This seemed to be due to size since it was observed for both anti-ICAM/3DNA and IgG/3DNA. Nevertheless, the lower blood level for targeted vs. untargeted formulations followed the 4L trend, due to fast lung targeting (Supplementary Figure S1), which was specific vs. IgG/3DNA or 3DNA. 2L anti-ICAM/3DNA also had lower blood levels and higher lung distribution compared to anti-ICAM Ab (Supplementary Figure S2), which is expected due to the role of size in clearance and increased avidity of multivalent NCs [7]. The specificity index of 2L anti-ICAM/3DNA, which takes into account the tissue-over-circulation levels for the targeted-over-untargeted formulations (Supplementary Figure S1) was similar to values reported for 4L counterparts [7].

**Figure 4:**
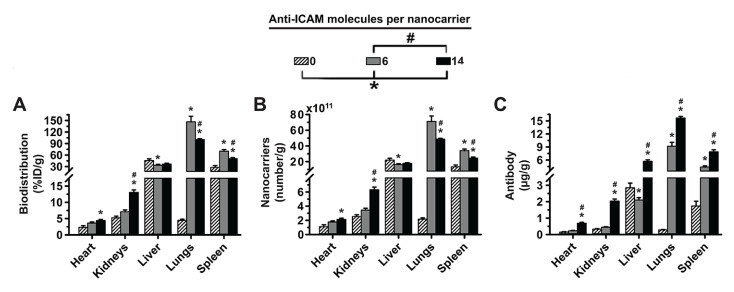

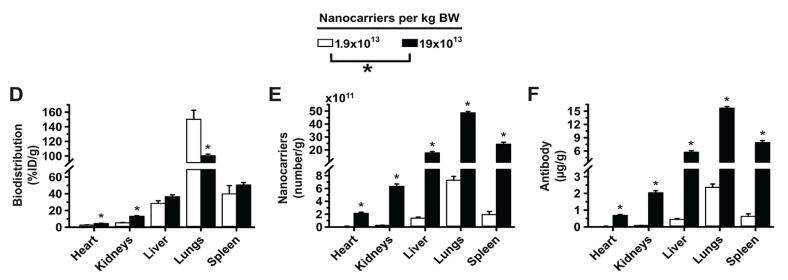
Effect of 2L anti-ICAM/3DNA targeting valency and dose concentration on biodistribution. C57BL/6 mice were IV injected with 2-layer ^125^I-anti-ICAM/3DNA at (**A**–**C**) different valencies (valency 0 = IgG/3DNA control), or (**D**–**F**) different dose concentrations. Organs’ radioactive content and weight were determined at 60 min to calculate: (**A**,**D**) % injected dose per gram of organ (%ID/g); (**B**,**E**) the number of nanocarriers per gram of organ; and (**C**,**F**) the antibody mass per gram of organ. Control ^125^I-IgG/3DNA is represented as targeting valency 0. Data are mean ± S.E.M (n ≥ 3). * Compares formulations with (**A**–**C**) 0 anti-ICAM/NC or (**D**–**F**) 1.9 × 10^13^ NCs/kg BW to those with higher valency or dose, respectively. # Compares formulations with (**A**–**C**) 6 anti-ICAM molecules/NC to those with greater valency; (*p* < 0.05).

Interestingly (Figure 4A), when we compared 2L anti-ICAM/3DNA with targeting valencies 6 vs. 14 Ab/NC (Table 1), we observed that increasing valency decreased lung and spleen distribution without impacting the liver. Consequently, the NC concentration in these organs showed a similar behavior (Figure 4B), while the Ab concentration increased for increasing valencies in all organs (Figure 4C). This is due to the difference in Ab loading of each NC being greater than the biodistribution change, just as for 4L formulations (Figure 2B–D). Increasing the dose concentration of 2L anti-ICAM/3DNA (Supplementary Table S1) also resulted in decreased lung targeting, without much impact on liver and spleen distribution (Figure 4D). As for the impact on the concentration of NCs (Figure 4E) and Ab (Figure 4F), increasing the dose concentration of 2L anti-ICAM/3DNA augmented these parameters for all organs, as found for 4L formulations (Figure 2F,G).

Intriguingly, we observed an opposite behavior for 2L vs. 4L formulations regarding the impact of targeting valency and dose concentration in lung biodistribution (compare Figure 2B,E to Figure 4A,D), where 2L behavior was more similar to that of previously published anti-ICAM polymeric nanoparticles [32]. Since the targeting valency of 2L 3DNA surpassed that of its 4L counterparts and that of previous polymeric nanoparticles [32], targeting saturation may be the reason for this difference. Notably, organs which received a small fraction of the 2L formulations showed enhanced accumulation with increasing valencies and dose concentrations, e.g., the heart. This suggests a similar role for valency for 2L and 4L formulations and might indicate that a saturation point was surpassed for 2L anti-ICAM/3DNA at valency 14 and dose 1.9 × 10^14^ NCs/kg BW. Saturating cell surface receptors in a concentration-dependent manner is a common finding for receptor-targeted nanocarriers, but saturation due to valency has been detected with more scarcity [40,41]. It is possible that this saturation was reached for the lungs but not other organs such as the heart, due to higher ICAM-1 expression and/or more extensive endothelial surface for binding [37], reflected in higher lung capacity to accumulate these NCs. Since the lungs receive full cardiac output as a first pass after IV injection, they act as a sink for these NCs and saturate first. Hence, the different behavior of 2L vs. 4L may not be due to size but rather different saturating capacities of the parametric values studied, further described in Section 3.5.

### 3.4. Significance of Targeting Valency and Dose Concentration of 2L Anti-ICAM/3DNA for Intercalating vs. Arm-Linked Drugs

**Figure 5:**
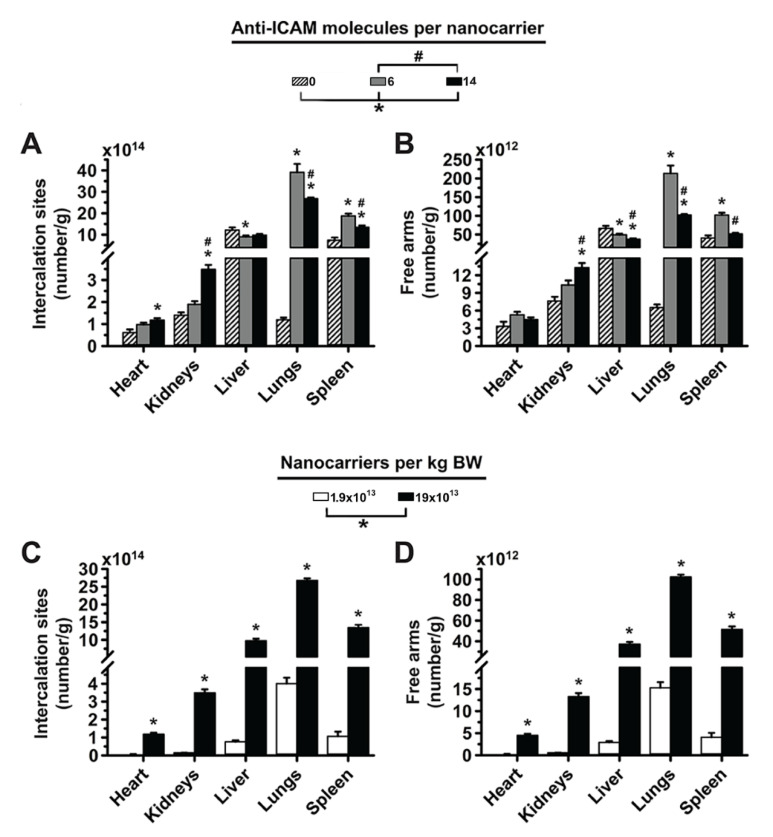
Effect of 2L anti-ICAM/3DNA targeting valency and dose concentration on the potential biodistribution of intercalating and arm-coupling drugs. 2-layer ^125^I-anti-ICAM/3DNA were IV injected into C57BL/6 mice at (**A**,**B**) different targeting valencies, or (**C**,**D**) different dose concentrations. Organs’ radioactive content and weight were determined at 60 min to calculate: (**A**,**C**) the number of DNA drug-intercalating sites per gram of organ; and (**B**,**D**) the number of outer arms free for drug coupling per gram of organ. Data are mean ± S.E.M. (n ≥ 3). * Compares formulations with (**A**,**B**) 0 anti-ICAM/NC or (**C**,**D**) 1.9 × 10^13^ NCs/kg BW to those with higher valency or dose, respectively. # Compares formulations with (**A**,**B**) 6 anti-ICAM molecules/NC to those with greater valency; (*p* < 0.05).

We then considered the biodistribution that 2L anti-ICAM/3DNA would provide for drugs (Figure 5). When increasing the targeting valency, the concentration of both intercalating sites and free arms decreased in the lungs, liver, and spleen, while increasing the dose concentration enhanced both these parameters in all organs. Compared to 4L formulations (Figure 3), this profile is similar in all aspects except for increased valency causing a reduction in the lung concentration of intercalating sites, which was the result of the saturation discussed above. For this reason, the decrease in NC arms free for drug coupling was more acute for 2L formulations, though the overall role of targeting valency and dose concentration in terms of drug biodistribution by 2L and 4L anti-ICAM/3DNA appear similar.

### 3.5. Multiparametric Comparison between 2L and 4L Anti-ICAM/3DNA Biodistribution

**Figure 6:**
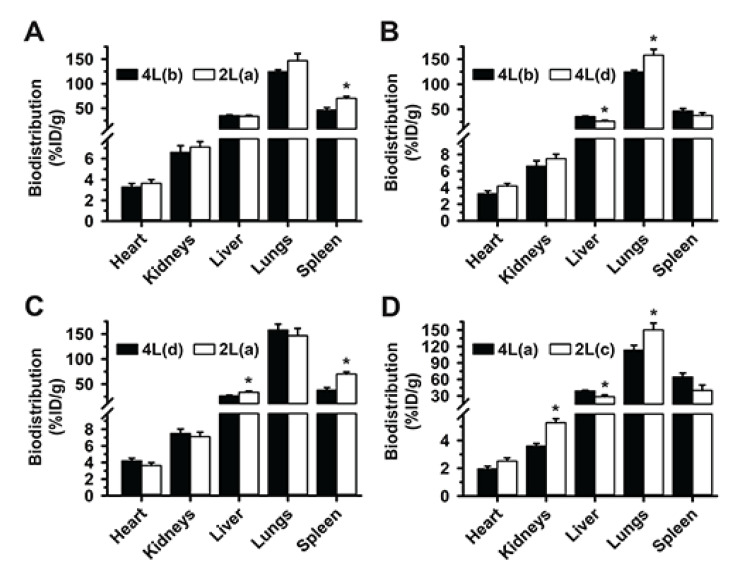

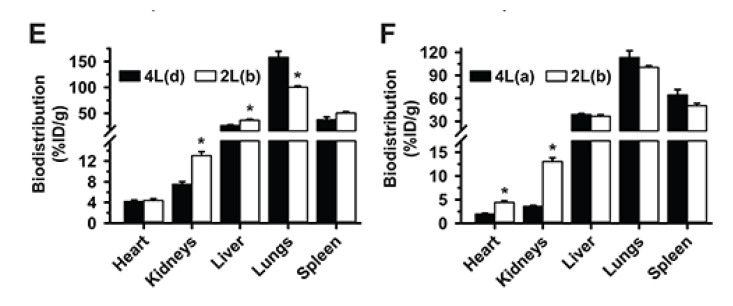
Multiparametric comparison between 2L and 4L anti-ICAM/3DNA biodistribution. ^125^I-anti-ICAM/3DNA were IV injected into C57BL/6 mice at different sizes, targeting valencies, and/or dose concentrations, so that for each comparison certain parameters were kept similar while others changed, to infer their impact hierarchy on lung targeting and biodistribution. Organs’ radioactive content and weight were determined at 60 min to calculate the % injected dose per gram of organ (% ID/g). All formulations and parametric values are in Supplementary Table S3. (**A**) Similar valency density, antibody dose, DNA dose, and total carrier surface, but different valency and number of nanocarriers. (**B**) Similar antibody dose, greater valency density for 4L(d), and lower DNA dose, total carrier surface, and number of carriers for 4L(d). (**C**) Similar antibody dose, greater valency density for 4L, lower DNA dose, total carrier surface, and number of nanocarriers for 4L. (**D**) Similar antibody dose, valency, and number of nanocarriers, greater valency density for 2L(c), and lower DNA dose, total carrier surface. (**E**) Greater valency density, antibody dose, DNA dose, total carrier surface, and number of nanocarriers for 2L(b); an oversaturating condition. (**F**) Similar valency, DNA dose and total carrier surface, although still greater antibody dose and number of nanocarriers for 2L(b). Data are mean ± S.E.M. (n ≥ 5). * Compares each pair of formulations shown (*p* < 0.05).

Data described above showed differences between 2L and 4L anti-ICAM/3DNA, but we could not assign these differences to size alone because other factors varied between the formulations tested. For instance, if the same number of NCs were used for 4L and 2L structures, the number of Abs per NC would vary between these formulations, or if the same number of Abs per NC were used then the Ab density on the NC surface would vary, etc. Hence, to clarify these relationships, we compared 2L and 4L formulations side-by-side by varying certain parameters while keeping others constant (Figure 6).

First, we compared valency 46 for 4L formulation to valency 6 for its 2L counterpart, both at 400 µg DNA/kg BW dose concentration, which had shown no signs of saturation. As shown in Supplementary Table S1 (2L*(a) vs. 4L(b))*, both formulations had similar Ab per NC surface (valency density), Ab dose, DNA dose, and NC surface area, representing the most equivalent among all 2L and 4L formulations. Differences were only in the Ab/NC valency (6 for 2L vs. 46 for 4L) and the number of NCs administered (9-fold lower for 4L). These formulations had statistically similar targeting to the lungs and biodistribution to all other organs, except the spleen where 2L anti-ICAM/3DNA was enhanced (Figure 6A). This indicated that within the tested ranges, size, absolute valency, and NC concentration were not predominant properties for biodistribution, in agreement with cell culture studies [42]. Properties with more predominant effect were those among the group of valency density, Ab dose administered, 3DNA dose administered, and total surface of the NCs injected, which were kept constant in this comparison. Yet, previous data had shown that valency density modulated biodistribution without varying the 3DNA dose administered or total surface area of all NCs injected (Figure 2 or Figure 4); hence, these properties would be less predominant than valency density and Ab dose injected.

Next, a comparison of formulations 4L(*b*) vs. 4L(*d*) (Figure 6B), which had similar Ab dose injected but different valency density (Supplementary Table S1), showed enhanced lung targeting and lower liver distribution for the formulation carrying higher valency density, although this formulation was administered at lower DNA dose, NC concentration, and total NC surface per injection. A similar trend was observed comparing formulations 2L(*a*) vs. 4L(*d*) (Figure 6C), which had similar Ab dose injected but different valency density (Supplementary Table S1), although this was not statistically significant in the lungs likely because 2L(*a*) had greater DNA dose, NC concentration, and total NC surface per injection, which may have compensated for the lower valency density. In fact, comparing formulations 2L(*c*) vs. 4L(*a*) which also had similar Ab dose injected, 2L(*c*) resulted in greater lung targeting and lower liver distribution, in agreement with its greater valency density, although the 2L formulation had a 10-fold lower DNA dose and total NC surface injected (Figure 6D).

Altogether, these data suggest that, within the tested range, the valency density is the most relevant parameter driving targeting; yet other parameters can affect the biodistribution profile achieved by modulating this overruling feature. For instance, 2L(*b*) formulation with similar valency density as the previous one (1273 Ab/µm^2^) but administered at 10-times higher Ab and DNA dose than 2L(*c*), had lower lung targeting and higher liver distribution than 4L(*d*) with 879 Ab/µm^2^ (Figure 6E), even though this 4L formulation had greater valency density than the one above (143 Ab/µm^2^). This suggests that the combination of the valency density and DNA dose for 2L(*b*) renders oversaturation in the main target (lung), as seen in Figure 4A. This was not observed for organs which received lower NC levels, the heart and kidneys, where biodistribution was enhanced for 2L(*b*). Oversaturation of the lungs vs. other organs has been discussed above. Since this phenomenon had also been observed in Figure 4A comparing formulations of the same size 2L(*a*) vs. 2L(*b*), lung saturation does not seem to associate with NC size. In fact, similar lung, liver, and spleen distribution were observed for 2L(*b*) and 4L(*a*)*,* which were more similar for most parameters compared to the previous 2L vs. 4L comparison discussed (Figure 6F).

### 3.6. Comparative Drug Delivery Capacity for 2L and 4L anti-ICAM/3DNA

**Figure 7:**
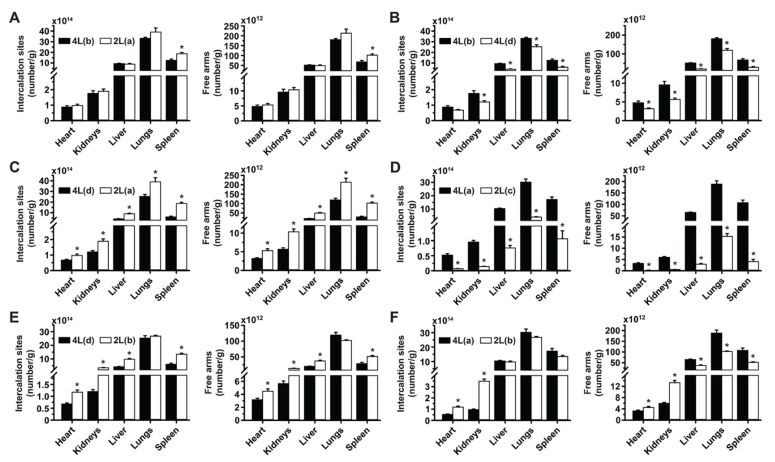
Comparative drug biodistribution capacity for 2L and 4L anti-ICAM/3DNA. ^125^I-anti-ICAM/3DNA were IV injected into C57BL/6 mice at different sizes, targeting valencies, and/or dose concentrations, so that for each comparison, certain parameters were kept similar while others were changed, to infer their impact hierarchy on lung targeting and biodistribution. Organs’ radioactive content and weight were determined at 60 min to calculate the number of DNA drug-intercalating sites per gram of organ (left plot) and the number of outer arms free for drug coupling per gram of organ (right plot). All formulations and parametric values are in Supplementary Table S3 and the relative comparisons shown here (A–F) are the same as those described in Figure 6. Data are mean ± S.E.M. (n ≥ 5). * Compares each pair of formulations shown (*p* < 0.05).

Finally, we examined the impact that size vs. other parametric variations of anti-ICAM/3DNA would have on the distribution of drugs that could be theoretically loaded. 2L and 4L formulations which were similar in most parameters (2L(*a*) and 4L(*b*)) were equivalent for biodistribution of drug-intercalating sites and arm-linked drugs, except in the spleen (Figure 7A), reflecting the biodistribution of these formulations (Figure 6A). Hence, the differences in size between 2L and 4L 3DNA did not play a major role in targeting/biodistribution at the organ level, for which both are similarly valuable.

Comparisons of 4L(*b*) vs. 4L(*d*) (Figure 7B), 4L(*d*) vs. 2L(*a*) (Figure 7C), and 4L(*a*) vs. 2L(*c*) (Figure 7D), which had shown increased lung targeting with increased valency density, rendered enhanced drug-intercalating sites and drug-linking arms depending on the injected dose concentration. This is because, although valency density more acutely rules the proportion of the injected dose that reaches the target (lungs) vs. clearance organs, the injected dose concentration more directly modifies the absolute amount of a drug reaching said tissues. This phenomenon did not depend on size, as the same trend was observed comparing formulations with the same or different sizes (Figure 7B or Figure 7C) and seemed proportional to the change in dose concentration independentof size (Figure 7C to Figure 7D). In all cases, the biodistribution of drug-linking arms appeared slightly more sensitive to parametric variations than the biodistribution of drug-intercalating sites.

For the comparison where the 2L formulation was oversaturating (2L(*b*) vs. 4L(*d*)*;* Figure 7E) and had lowered lung targeting, the greater dose concentration employed compensated for this saturation and resulted in a similar lung biodistribution for drug-intercalating sites and drug-linking arms as the 4L formulation. However, the greater dose concentration caused the 2L formulation to render much higher drug biodistribution in the liver and spleen. Comparing the same 2L(*b*) formulation to 4L(*a*), so that other parametric differences were less acute, still showed a greater targeting of the lung over other organs for the 4L formulation in terms of drug-intercalating sites and drug-linking arms (Figure 7F). Hence, overcompensating for targeting deficiencies by increasing the dose administered for oversaturating valency densities may not be a good approach as this seems to negatively influence lung targeting.

## 4. Conclusions

**Figure 8:**
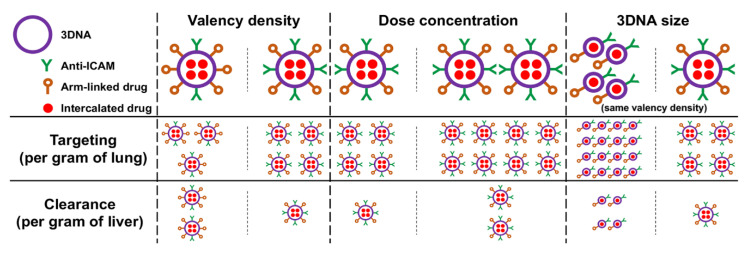
Role of design and administration parameters in the biodistribution of anti-ICAM/3DNA and carrier drugs. The schematic shows the contribution of (i) valency density (the number of targeting antibodies per nanocarrier (NC) surface area), (ii) anti-ICAM/3DNA dose concentration in the injected dose, and (iii) anti-ICAM/3DNA size on the biodistribution of NCs and respective intercalating or arm-linked drugs, both for the specific target site (the lungs) and a main clearance organ (liver). While the proportions shown do not exactly match the findings, they summarize well the conclusions found.

Both 2L and 4L anti-ICAM/3DNA had good lung specificity, achieved at valencies much lower than for previous polymeric nanoparticles [32], likely because Ab annealing on 3DNA outer arms provides greater flexibility and/or target accessibility. This explains why a saturating formulation was found for 3DNA but not previous nanoparticles [32], although this will depend on ICAM-1 levels, e.g., known overexpression in disease-associated inflammation may further enhance lung delivery before reaching saturation. This is an important point since surpassing lung saturation would re-direct 3DNA to other organs, which can be an advantage or disadvantage depending on whether lung targeting alone or lung + systemic distribution is needed. As per NC size, 2L and 4L did not render any major difference in anti-ICAM/3DNA biodistribution (Figure 8), and this should not affect uptake by cells either due to the size-permissive, ICAM-1 endocytic pathway [22]. However, smaller 2L formulations may be beneficial when targeting size-sensitive routes [34,43,44,45], when the absolute number of NCs per cell would be relevant for effects [46] or given the simpler synthesis of 2L vs. 3L 3DNA. In addition, we demonstrated that valency density is more relevant for targeting than absolute valency (Figure 8), in agreement with our previous study in cell cultures [42]. Since this had been observed using polymer NCs bearing adsorbed anti-ICAM, this prominent role stands across carriers and coupling modes. Also, in this system and focusing on lung targeting, valency density was more relevant than dose concentration (Figure 8) and may be considered the primary parameter to modulate site-specific delivery of drugs which can be highly toxic at off-target sites, as is the case for harsh chemotherapeutics used for lung cancer [47]. This parameter is less relevant in cases where patients may benefit from lung and systemic treatment, such as enzyme replacement for type B Niemann–Pick disease, where the lung is the predominant but not the only organ involved [21]. Both valency and dose concentration could be adjusted to achieve the required level of lung specificity and absolute dose delivered to the lungs. In addition, our data showed that the biodistribution of arm-linked drugs would be more sensitive to tuning 3DNA parameters than intercalating drugs (Figure 8) and different drugs would differently benefit from varying these design parameters. For instance, increasing the targeting valency would reduce the number of arms free for drug coupling, but this will not apply to intercalating drugs (Figure 8). Hence, valency must be more carefully balanced for arm-linked drugs to achieve sufficient lung specificity while carrying enough molecules of a therapeutic cargo, and a possible solution would consist of attaching multiple drug molecules to the same arm.

In summary, the knowledge obtained in this study is relevant in selecting which parameter(s) of anti-ICAM/3DNA should be varied depending on the drug type and application pursued, which shall guide future investigations focusing on specific drugs for particular lung maladies. In this regard, although no adverse effects were observed in this study, future works will aim to assess the potential toxicity of anti-ICAM/3DNA. Anti-ICAM has been tested in clinical trials for other applications without major side effects [48]. 3DNA has been designed to avoid CpG sequences and minimize recognition by Toll-like receptors and activation of immune cascades [49], which could be further avoided by using non-natural nucleic acids [50]. Other important items are the suitability of 3DNA for clinical applications in terms of fabrication and cost, where 3DNA represents an interesting strategy with balanced advantages and disadvantages, as for most other NCs. For instance, oligonucleotides used to build 3DNA may be more expensive than more classical polymers used for drug delivery. However, their in vitro synthesis using either PCR or synthetic assembly has become highly precise and cost-effective [51], an example of which is the recent worldwide release of anti-COVID-19 vaccines based on nucleic acids [52]. Self-assembly by simple annealing of oligos into 3DNA structures renders ≥98% yield and is highly reproducible and uniform (Code Biotherapeutic internal information), unlike more classical polymeric NCs [53]. Additionally, 3DNA fabrication does not require the use of solvents that may harm both the therapeutic cargo and patient health, along with causing environmental concerns [53]. These advantages, together with high targeting specificity and sequence-based tunability of 3DNA properties seen in this study, suggest that a speculative higher fabrication cost for 3DNA may be well compensated by these advantages, representing a valuable drug delivery system.

## Figures and Tables

**Table 1:** Characterization of 3DNA formulations.

Formulation	Targeting Ab Valency (Ab/NC)	Targeting Ab Density (Ab/µm^2^)	Mean Diameter (nm)	PDI	ζ-Potential (mV)
**4L 3DNA**					
IgG control (i)	0	0	181.2 ± 5.1	0.23	−42.7 ± 0.5
Anti-ICAM F1	13	142.8	160.0 ± 3.5	0.18	−39.1 ± 0.8
Anti-ICAM F2	46	505.5	179.5 ± 5.7	0.25	−43.6 ± 0.1
Anti-ICAM F3	80	879.1	196.6 ± 3.5	0.20	−45.0 ± 0.1
**2L 3DNA**					
IgG control (ii)	0	0	114.4 ± 3.1	0.34	−38.6 ± 2.1
Anti-ICAM F1	6	545.5	113.0 ± 4.4	0.35	−36.9 ± 1.8
Anti-ICAM F2	14	1272.7	120.4 ± 3.0	0.29	−35.3 ± 0.3

Ab = antibody; F = formulation; NC = nanocarrier; PDI = polydispersity index. Data are mean ± S.E.M. (i) 4L IgG/3DNA control contained 0 anti-ICAM molecules (targeting valency 0) and 46 IgG molecules/NC (505.5 Ab/µm^2^); (ii) 2L IgG/3DNA control contained 0 anti-ICAM molecules (targeting valency 0) and 6 IgG molecules/NC (545.5 Ab/µm^2^).

## Data Availability

Data will be made available upon request.

## Supplementary Materials

The following supporting information can be downloaded at: https://www.mdpi.com/article/10.3390/pharmaceutics14071496/s1, Table S1: Parametric values of all anti-ICAM/3DNA injections; Table S2: Blood distribution for anti-ICAM/3DNA and control formulations; Table S3: Organ distribution for anti-ICAM/3DNA and control formulations; Figure S1: Biodistribution of 2L anti-ICAM/3DNA; Figure S2: Biodistribution of 2L anti-ICAM/3DNA vs. anti-ICAM.
