# Supplementary material for: Tuning Design Parameters of ICAM-1-Targeted 3DNA Nanocarriers to Optimize Pulmonary Targeting Depending on Drug Type
Source: Pharmaceutics. 2022 Jul 19;14(7):1496. doi: 10.3390/pharmaceutics14071496 (PMC9316040; doi:10.3390/pharmaceutics14071496)
Supplement: Supplementary file 1 [file pharmaceutics-14-01496-s001.zip › pharmaceutics-1726162-supplementary.pdf]

pharmaceutics

Supplementary Materials: Tuning Design Parameters of ICAM-1-Targeted 3DNA Nanocarriers to Optimize Pulmonary Targeting Depending on Drug Type

Nikša Roki, Melani Solomon, Jessica Bowers, Lori Getts, Robert C. Getts and Silvia Muro

Table S1. Parametric values of all anti-ICAM/3DNA injections.

Formulation	Properties & Parameters							
	Valency (Ab/NC)	Valency density (Ab/μm² NC)	Ab dose (μg/kg BW)	DNA dose (μg/kg BW)	3DNA surface (μm²/kg BW)	NCs/kg BW	Intercalation Sites/kg BW	Free Arms/BW
**4L 3DNA**								
Anti-ICAM *(a)*	13	142.8	69	400	19E+11	21.5E+12	1.1E+17	6.6E+15
Anti-ICAM *(b)*	46	505.5	249	400	19E+11	21.5E+12	1.1E+17	5.8E+15
Anti-ICAM *(c)*	80	879.1	415	400	19E+11	21.5E+12	1.1E+17	5.0E+15
Anti-ICAM *(d)*	80	879.1	249	240	12E+11	12.9E+12	6.4E+16	3.0E+15
Anti-ICAM *(e)*	80	879.1	42	40	1.9E+11	2.2E+12	1.1E+16	5.0E+14
**2L 3DNA**								
Anti-ICAM *(a)*	6	545.5	250	400	22E+11	194E+12	1.1E+17	5.8E+15
Anti-ICAM *(b)*	14	1272.7	625	400	22E+11	194E+12	1.1E+17	4.1E+15
Anti-ICAM *(c)*	14	1272.7	63	40	2.2E+11	19.4E+12	1.1E+16	4.1E+14

Ab = antibody; BW = body weight, NC = nanocarrier.

Table S2. Blood distribution for anti-ICAM/3DNA and control formulations.

Formulation	% ID in Blood				
	1 min	5 min	15 min	30 min	60 min
**4L 3DNA**					
No Ab	53.3±1.9	4.7±0.7	14.3±0.7	2.2±0.2	7.3±2.1
IgG control $_{(i)}^{21.5}$	41.9±2.9	17.2±0.4	31.2±1.4	23.5±1.0	28.6±2.2
Anti-ICAM $_{13}^{21.5}$	21.8±1.9	3.1±0.1	1.7±0.3	2.4±0.1	2.3±0.2
Anti-ICAM $_{46}^{21.5}$	17.6±2.9	4.0±0.3	2.7±0.6	3.3±0.4	3.0±0.2
Anti-ICAM $_{80}^{21.5}$	11.2±1.0	3.6±0.1	4.3±0.3	3.4±0.5	2.5±0.2
Anti-ICAM $_{80}^{12.9}$	13.4±1.5	ND	3.5±0.3	3.1±0.4	2.6±0.1
Anti-ICAM $_{80}^{2.2}$	20.7±4.3	5.3±0.8	3.3±0.6	2.6±0.5	2.7±0.3
**2L 3DNA**					
No Ab	57.8±7.0	28.5±2.6	12.4±1.3	ND	8.2±0.6
IgG control $_{(ii)}^{194}$	55.3±1.9	33.8±0.9	30.6±2.1	ND	34.2±1.4
Anti-ICAM $_{6}^{194}$	31.2±2.9	4.7±0.9	4.2±0.6	3.1±0.3	2.5±0.3
Anti-ICAM $_{14}^{194}$	18.1±2.5	6.1±0.3	4.5±0.2	4.0±0.3	3.8±0.1
Anti-ICAM $_{14}^{19.4}$	25.0±4.1	4.5±0.9	2.7±0.0	2.8±0.5	2.2±0.3

Ab = antibody; % ID = percentage of the injected dose; ND = not determined. Data are mean ± S.E.M. The subscript on the antibody name represents the targeting valency (antibody molecules per nanocarrier) shown in Table 1. The superscript is the dose concentration (number of nanocarriers per kg of body weight). All values must be multiplied by 10^{12}. (i) 4L IgG/3DNA control contained 0 anti-ICAM molecules (targeting valency 0) and 46 IgG molecules/NC. (ii) 2L IgG/3DNA control contained 0 anti-ICAM molecules (targeting valency 0) and 6 IgG molecules/NC.

Pharmaceutics **2022**, *14*, 1496. https://doi.org/10.3390/pharmaceutics14071496 www.mdpi.com/journal/pharmaceutics

Table S3. Organ distribution for anti-ICAM/3DNA and control formulations.

Formulation	% ID in Organ				
	Heart	Kidneys	Liver	Lungs	Spleen
**4L 3DNA**					
No Ab	0.1±0.0	3.2±0.7	20.8±1.3	0.5±0.1	1.0±0.1
IgG control $_{(i)}^{21.5}$	0.1±0.0	1.1±0.1	50.2±2.0	0.4±0.1	2.2±0.1
Anti-ICAM $_{13}^{21.5}$	0.2±0.0	1.3±0.1	45.8±1.3	14.5±1.1	4.7±0.4
Anti-ICAM $_{46}^{21.5}$	0.4±0.0	2.6±0.3	40.2±1.7	18±1.0	3.5±0.3
Anti-ICAM $_{80}^{21.5}$	0.5±0.0	3.2±0.3	35.5±0.8	18.6±0.5	3.0±0.2
Anti-ICAM $_{80}^{12.9}$	0.5±0.0	2.8±0.2	32.7±2.4	21.1±1.4	3.5±0.6
Anti-ICAM $_{80}^{2.2}$	0.3±0.0	1.9±0.0	39.2±1.1	18.4±0.8	3.6±0.3
**2L 3DNA**					
No Ab	0.2±0.0	2.4±0.2	20.6±1.1	0.5±0.1	1.3±0.1
IgG control $_{(ii)}^{194}$	0.2±0.1	1.6±0.2	46.2±1.2	0.6±0.0	1.8±0.2
Anti-ICAM $_{6}^{194}$	0.4±0.0	2.2±0.1	37.5±1.1	18.4±0.7	4.6±0.2
Anti-ICAM $_{14}^{194}$	0.5±0.0	4.3±0.2	39.2±2.3	13±0.3	3.5±0.1
Anti-ICAM $_{14}^{19.4}$	0.3±0.0	1.9±0.1	34.3±2.3	19.6±1.3	3.3±0.6

Ab = antibody; % ID = percentage of the injected dose; ND = not determined. Data are mean ± S.E.M. The subscript on the antibody name represent the targeting valency (antibody molecules per nanocarrier) shown in Table 1. The superscript is the dose concentration (number of nanocarriers per kg of body weight). All values must be multiplied by 10^{12}. (i) 4L IgG/3DNA control contained 0 anti-ICAM molecules (targeting valency 0) and 46 IgG molecules/NC. (ii) 2L IgG/3DNA control contained 0 anti-ICAM molecules (targeting valency 0) and 6 IgG molecules/NC.

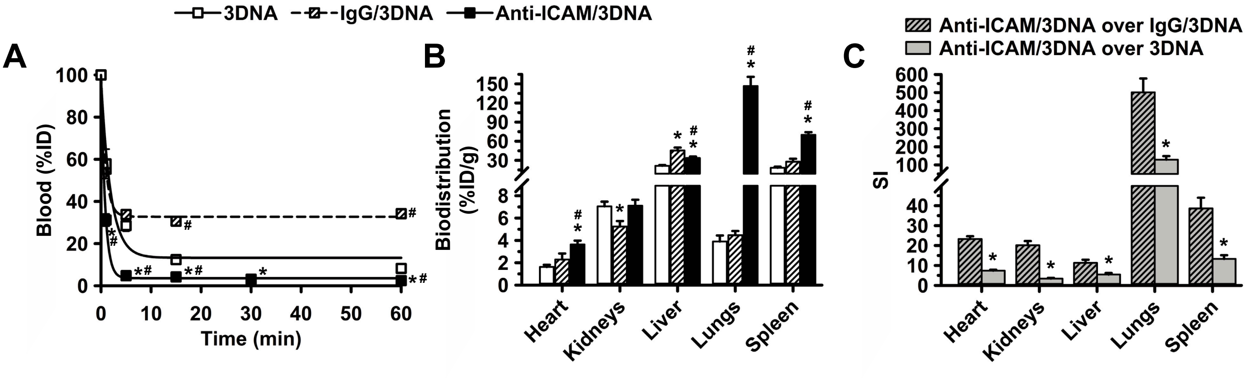

Figure S1. Biodistribution of 2L anti-ICAM/3DNA. ^{125}I-anti-ICAM/3DNA, control ^{125}I-IgG/3DNA, or control ^{125}I-3DNA were i.v. injected in C57BL/6 mice. Organs and blood radioactive content and weight were determined at (A) the indicated times or (B,C) 60 min after injection, to calculate: (A) the percentage of the injected dose in blood (% ID), (B) % ID per gram of organ (% ID/g) to compare organs of different weight, and (C) the specificity index (SI), which represents the organ-to-blood ratio of the targeted formulation over the non-targeted formulation (see Experimental Section). Data are mean ± S.E.M. * Compares either antibody/3DNA vs. 3DNA alone; # compares anti-ICAM/3DNA vs. IgG/3DNA; ($p < 0.05$ by Student's *t*-test).

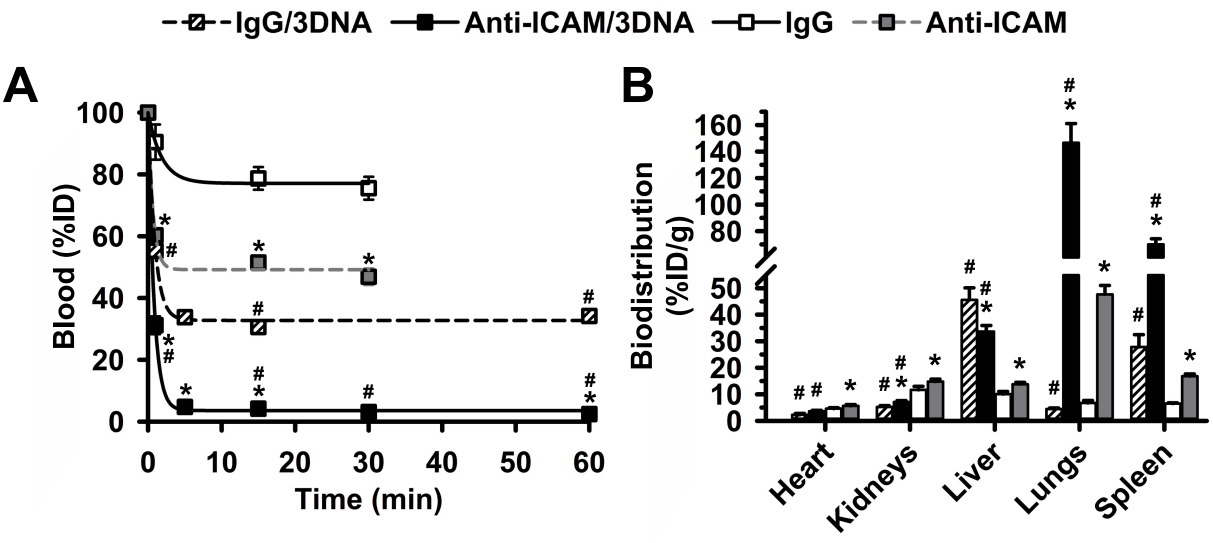

Figure S2. Biodistribution of 2L anti-ICAM/3DNA *vs.* anti-ICAM. ^{125}I-anti-ICAM/3DNA, control ^{125}I-IgG/3DNA, or corresponding antibodies (^{125}I-IgG or ^{125}I-anti-ICAM) were i.v. injected in C57BL/6 mice. Organs and blood radioactive content and weight were determined at (A) the indicated times or (B) 60 min after injection for 2L 3DNA formulations and 30 min after injection for antibodies, to calculate: (A) the percentage of the injected dose in blood (% ID), and (B) % ID per gram of organ (% ID/g) to compare organs of different weight. Data are mean ± S.E.M. * Compares anti-ICAM formulations *vs.* corresponding IgG counterparts; # compares antibody/3DNA vs. corresponding Ab counterparts; ($n \geq 3$; $p < 0.05$ by Student's t-test).
